# A randomized double-blind multi-center trial of hydrogen water for Parkinson’s disease: protocol and baseline characteristics

**DOI:** 10.1186/s12883-016-0589-0

**Published:** 2016-05-12

**Authors:** Asako Yoritaka, Takashi Abe, Chigumi Ohtsuka, Tetsuya Maeda, Masaaki Hirayama, Hirohisa Watanabe, Hidemoto Saiki, Genko Oyama, Jiro Fukae, Yasushi Shimo, Taku Hatano, Sumihiro Kawajiri, Yasuyuki Okuma, Yutaka Machida, Hideto Miwa, Chikako Suzuki, Asuka Kazama, Masahiko Tomiyama, Takeshi Kihara, Motoyuki Hirasawa, Hideki Shimura, Nobutaka Hattori

**Affiliations:** Department of Neurology, Juntendo University Koshigaya Hospital, Fukuroyama 560, Koshigayashi, Saitama 343-0032 Japan; Department of Neurology, Juntendo University School of Medicine, Tokyo, Japan; Department of Neurology, Abe Neurological Clinic, Iwate, Japan; Department of Neurology and Gerontology, Iwate Medical University, Iwate, Japan; Department of Neurology, Research Institute for Brain and Blood Vessels-Akita Hospital, Akita, Japan; Department of Pathophysiological Laboratory Sciences, Nagoya University Graduate School of Medicine, Aichi, Japan; Brain and Mind Research Center, Nagoya University Graduate School of Medicine, Aichi, Japan; Department of Neurology, Kitano Hospital, The Tazuke Kofukai Medical Research Institute, Osaka, Japan; Department of Neurology, Fukuoka University, Fukuoka, Japan; Department of Neurology, Juntendo University Shizuoka Hospital, Shizuoka, Japan; Department of Neurology, Juntendo University Nerima Hospital, Tokyo, Japan; Department of Diagnostic Radiology, Department of Molecular Medicine and Surgery, Karolinska University Hospital, Karolinska Institute, Stockholm, Sweden; Nozomi Hospital, Saitama, Japan; Department of Neurology, Aomori Prefectural Central Hospital, Aomori, Japan; Department of Neurology, Rakuwakai Otowa Rehabilitation Hospital, Kyoto, Japan; Department of Neurology, Tokyo Rinkai Hospital, Tokyo, Japan; Department of Neurology, Juntendo University Urayasu Hospital, Chiba, Japan

**Keywords:** Hydrogen, oxidative stress, Parkinson’s disease, randomized double-blind placebo-controlled multicenter trial

## Abstract

**Background:**

Our previous randomized double-blind study showed that drinking hydrogen (H_2_) water for 48 weeks significantly improved the total Unified Parkinson’s Disease Rating Scale (UPDRS) score of Parkinson’s disease (PD) patients treated with levodopa. We aim to confirm this result using a randomized double-blind placebo-controlled multi-center trial.

**Methods:**

Changes in the total UPDRS scores from baseline to the 8^th^, 24^th^, 48^th^, and 72^nd^ weeks, and after the 8^th^ week, will be evaluated. The primary endpoint of the efficacy of this treatment in PD is the change in the total UPDRS score from baseline to the 72^nd^ week. The changes in UPDRS part II, UPDRS part III, each UPDRS score, PD Questionnaire-39 (PDQ-39), and the modified Hoehn and Yahr stage at these same time-points, as well as the duration until the protocol is finished because additional levodopa is required or until the disease progresses, will also be analyzed. Adverse events and screening laboratory studies will also be examined. Participants in the hydrogen water group will drink 1000 mL/day of H_2_ water, and those in the placebo water group will drink normal water. One-hundred-and-seventy-eight participants with PD (89 women, 89 men; mean age: 64.2 [SD 9.2] years, total UPDRS: 23.7 [11.8], with levodopa medication: 154 participants, without levodopa medication: 24 participants; daily levodopa dose: 344.1 [202.8] mg, total levodopa equivalent dose: 592.0 [317.6] mg) were enrolled in 14 hospitals and were randomized.

**Discussion:**

This study will confirm whether H_2_ water can improve PD symptoms.

**Trial registration:**

UMIN000010014 (February, 13, 2013)

## Background

In patients with Parkinson’s disease (PD), the pharmacologic replacement of dopamine and other antiparkinsonian drugs has been used for symptomatic therapy. However, none of these drugs stop or lessen the dopaminergic neuronal degeneration or the progression of the disease. Findings of increased iron and lipid peroxidation and decreased levels of reduced glutathione in the substantia nigra strongly suggest that enhanced oxidative stress is involved in the pathogenesis of PD [1, 2]. Thus, antioxidant therapies might slow the progression of PD. Molecular hydrogen (H_2_) has recently been highlighted as a therapeutic and preventive antioxidant. Since the first publication [3], more than 150 papers have confirmed the efficacy of H_2_ in various animal models [4]. H_2_-water reduced dopaminergic neuronal cell loss in a 1-methyl-4-phenyl-1,2,3,6-tetrahydropyridine (MPTP) mouse model [5] as well as 6-hydroxydopamine did [6]. Our previous randomized double-blind study has shown that drinking 1,000 mL of H_2_-water for 48 weeks significantly improved (*p* < 0.05) the total Unified Parkinson’s Disease Rating Scale (UPDRS) scores of patients with PD who were being treated with levodopa [7]. In the present study, we aimed to confirm these results by conducting a longer and more large-scale trial that also included patients who were not being treated with levodopa. Here, we present the design and the baseline characteristics of participants already enrolled in this study.

## Methods

A placebo-controlled, randomized, double-blind, parallel-group (1:1) clinical multi-center trial was organized by the Department of Neurology of Juntendo University School of Medicine in accordance with Consolidated Standards of Reporting Trials (CONSORT) guideline. Fourteen hospitals are involved as trial centers. This trial was advertised in posters and on homepages of our clinic, and participants had to declare their intentions to participate voluntarily. The inclusion criteria required that the participants have a diagnosis of PD according to the United Kingdom Brain Bank criteria [8]. All the participants should have a modified Hoehn and Yahr staging (H & Y stage) in the on-phase between 1 and 4. For 8 weeks prior to establishing the baseline, the participants’ antiparkinsonian drugs were not changed. None of the participants have dementia (MMSE < 25) or dysphagia for water. Outpatients are preferred over admitted patients. The participants are to be older than 20 years. The exclusion criteria included the following: parkinsonism due to diseases other than PD, the presence of other serious diseases, malignant tumor(s), or adverse events caused by drugs.

The clinical study is registered at UMIN clinical trial registry (UMIN-CTR) UMIN000010014 (February 13, 2013). The Ethics Committee of the Juntendo University School of Medicine approved this study in February 2013, as did the ethics committees of other centers, and all participants provided signed informed consent forms.

### Randomization and blinding

The enrolled participants have been assigned using a stratified randomization method according to their age and if they were receiving levodopa. The assignments were made by C.S. The participants and those assessing outcomes will remain blinded until all the participants have finished the protocol.

### Procedures

On a daily basis, the participants will drink 1,000 mL of saturated H_2_-water containing 5 mM of dissolved H_2_ (using Hydrogen 7.0, supplied by Ecomo International Co., Ltd. [Fukuoka, Japan]; patent No: PCT/JP2011/063601) for 72 weeks. The placebo water is saturated with N_2_. The water is contained in a 500-mL plastic bottle, and the participants will drink 2 bottles per day. The participants will drink the water within 3 h of opening the cap, because H_2_ evaporates. The bottles of H_2_-water or placebo water will be sent to the participants’ home every week.

**Fig. 1:**
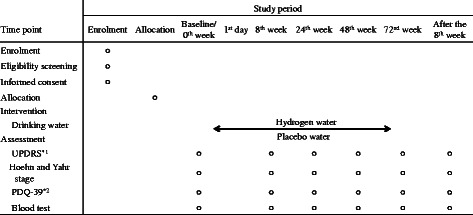
Schedule of enrolment, intervention, and assessment. Study period of enrolment, allocation, and baseline may coincide. It is approximately 1 week to 10 days from allocation to the first day to start shipping the water. *1: Unified Parkinson’s Disease Rating Scale. *2: Parkinson’s Disease Questionnaire-39

The schedule of the study is shown in Fig. 1. Changes in the total UPDRS scores from baseline to the 8^th^, 24^th^, 48^th^, and 72^nd^ weeks, and after the 8^th^ week, are evaluated. The primary endpoint of the efficacy of this treatment in PD is the change in the total UPDRS score from baseline to the 72^nd^ week.

The changes in UPDRS part II, UPDRS part III, each UPDRS score, PD Questionnaire-39 (PDQ-39), and the H & Y stage at these same time-points, as well as the duration until the protocol is finished because additional levodopa is required or until the disease progresses, will also be analyzed. Adverse events and screening laboratory parameters (total protein, albumin, alkaline phosphatase, aspartate transaminase, alanine transaminase, serum urea nitrogen, calcium, chloride, creatinine, glucose, lactate dehydrogenase, potassium, sodium, creatinine kinase, uric acid, choline esterase, LDL-cholesterol, HDL-cholesterol, and triglyceride levels) will also be examined.

### Statistical analysis

We calculated that a minimum of 95.4 participants need to be enrolled to detect a 5-point difference in the UPDRS scores between the 2 groups, with a standard deviation of the mean difference of 10, and a 2-sided alpha level of 0.05 and 80 %. Assuming a 35 % dropout, 96 participants will be required in total. A period of 72 weeks’ follow-up is a relatively long-term clinical trial in PD; hence, we assumed a 35 % dropout rate.

Variations in the endpoints between the baseline value and treatment assessment points will be compared between the groups, using a *t*-test or a Mann–Whitney U-test. The statistical tests are 2-sided, and the significance level is set at 0.05. The number of participants in the treatment and placebo groups who drop out due to disease progression will be analyzed using Kaplan − Meier curves and the log-rank test. Subgroup analysis (sex, and treatment with or without levodopa) will be performed.

### Enrollment and data collection

One-hundred-and-seventy-eight Japanese participants with PD have already been enrolled in 14 hospitals and have been randomized, between April 1, 2013 and September 30, 2015, at their baseline visit. The participants will be followed-up for 80 weeks. The baseline characteristics are shown in Table 1. Despite extensive efforts to enroll more participants, the expected number of participants was not met, as it is difficult for some patients to consume 1000 mL water daily.

**Table 1:** The baseline characteristics of the study participants

	Mean	Std. Deviation
Age	64.2	9.2
Disease duration	6.8	4.5
Sex (male/female) n	89	89
Wearing off (+/-) n	58	120
Dyskinesia (+/-) n	42	136
Levodopa mg	344.1	202.8
Levodopa (+/-) n	154	24
Dopamine agonist levodopa equivalent dose (mg) [12]	166.3	121.1
Total levodopa equivalent dose (mg) [12]^a^	592.0	317.6
Unified Parkinson’s Disease Rating Scale		
Part I	0.7	1.2
Part II	5.6	3.9
Part III	15.7	8.4
Part IV	1.7	2.2
Total	23.7	11.8
Modified Hoehn and Yahr stage	2.1	0.6
Parkinson’s disease Questionnaire-39		
Mobility	10.4	9.1
Activities of daily living	5.2	5.1
Emotional well-being	6.0	4.4
Stigma	3.2	2.8
Social support	1.4	1.9
Cognitions	4.0	3.0
Communication	1.7	2.2
Body discomfort	2.5	2.6
Total	34.4	24.2

^a^: not including istradefylline and zonisamide

## Discussion

Fujita *et al.* have indicated that the intake of H_2_-water, even after MPTP administration, reduces neurotoxic damage [5]. The findings of our previous study [7] on PD patients are in agreement with the previous results that were obtained in animal models.

Antioxidant supplements that are considered medicinal products should undergo sufficient evaluation before marketing, as they might be harmful at high doses [9]. H_2_ selectively reduces •OH radicals, but not O_2_^**−**^•, H_2_O_2_, or NO• [4]. It is expected that prolonged application of H_2_ will have no or little adverse effects in chronic diseases. The effects of H_2_ could be mediated by modulating activities and expression of various molecules, such as Lyn, ERK, p38, JNK, ASK1, Akt, GTP-Rac1, iNOS, Nox1, NF-κB, p65, Iκba, STST3, NFATc1, c-Fos, and ghrelin [10]. Iuchi et al. proposed a hypothetical model in which H_2_ is linked to the modulation of Ca^2+^ signal transduction and the nuclear factor of activated T cells (NFAT) pathway via oxidized phospholipid species [11].

Our previous trial was the first randomized double-blind study of H_2_-water in patients with PD [7]. H_2_-water exhibited no adverse effects at a dose of 1000 mL/day in PD subjects receiving levodopa treatment. The results of the previous study will be confirmed in this longer and larger-scale study that includes patients who are not medicated with levodopa. This study will confirm the safety and tolerability of H_2_-water and if H_2_-water can improve PD symptoms.

## Ethics approval

A randomized double-blind multi-center trial of hydrogen water for Parkinson’s disease was approved by the Ethics Committee of the Juntendo University School of Medicine, and the ethics committees of other centers.

## Consent for publication

Not applicable.

## Availability of data and material

Not applicable.

## Abbreviations

CONSORT: Consolidated Standards of Reporting Trials
H_2_: hydrogen
H & Y: modified Hoehn and Yahr staging
MPTP: 1-methyl-4-phenyl-1,2,3,6-tetrahydropyridine
NFAT: nuclear factor of activated T cells
PD: Parkinson’s disease
PDQ-39: Parkinson’s disease Questionnaire-39
UPDRS: Unified Parkinson’s Disease Rating Scale

## References

[1] Dexter DT, Wells FR, Agid F, Lees AJ, Jenner P, Marsden CD (1987). Increased nigral iron content in postmortem Parkinsonian brain. Lancet..

[2] Yoritaka A, Hattori N, Uchida K, Tanaka M, Stadtman ER, Mizuno Y (1996). Immunohistochemical detection of 4-hydroxynonenal protein adducts in Parkinson disease. Proc Natl Acad Sci USA.

[3] Ohsawa I, Ishikawa M, Takahashi K (2007). Hydrogen acts as a therapeutic antioxidant by selectively reducing cytotoxic oxygen radicals. Nat Med..

[4] Ohta S (1820). Molecular hydrogen is a novel antioxidant to efficiently reduce oxidative stress with potential for the improvement of mitochondrial diseases. Biochim Biophys Acta..

[5] Fujita K, Seike T, Yutsudo N (2009). Hydrogen in drinking water reduces dopaminergic neuronal loss in the 1-methyl-4-phenyl-1,2,3,6-tetrahydropyridine mouse model of Parkinson’s disease. PLoS One..

[6] Fu Y, Ito M, Fujita Y (2009). Molecular hydrogen is protective against 6-hydroxydopamine-induced nigrostriatal degeneration in a rat model of Parkinson’s disease. Neurosci Lett..

[7] Yoritaka A, Takanashi M, Hirayama M, Nakahara T, Ohta S, Hattori N (2013). Pilot study of H2 therapy in Parkinson’s disease: A randomized double-blind placebo-controlled trial. Mov Disord..

[8] Hughes AJ, Daniel SE, Kilford L, Lees AJ (1992). Accuracy of clinical diagnosis of idiopathic Parkinson’s disease: a clinico-pathological study of 100 cases. J Neurol Neurosurg Psychiatry..

[9] Bjelakovic G, Nikolova D, Gluud LL, Simonetti RG, Gluud C (2007). Mortality in randomized trials of antioxidant supplements for primary and secondary prevention: systematic review and meta-analysis. JAMA..

[10] Ichihara M, Sobue S, Ito M, Ito M, Hirayama M, Ohno K (2015). Beneficial biological effects and the underlying mechanisms of molecular hydrogen—comprehensive review of 321 original articles. Med Gas Res..

[11] Iuchi K, Imoto A, Kamimura N (2016). Molecular hydrogen regulates gene expression by modifying the free radical chain reaction-dependent generation of oxidized phospholipid mediators. Sci Rep..

[12] Tomlinson CL, Stowe R, Patel S, Rick C, Gray R, Clarke CE (2010). Systematic review of levodopa dose equivalency reporting in Parkinson’s disease. Mov Disord..

