# Erratum to: A randomized double-blind multi-center trial of hydrogen water for Parkinson’s disease: protocol and baseline characteristics

**DOI:** 10.1186/s12883-017-0817-2

**Published:** 2017-02-20

**Authors:** Asako Yoritaka, Takashi Abe, Chigumi Ohtsuka, Tetsuya Maeda, Masaaki Hirayama, Hirohisa Watanabe, Hidemoto Saiki, Genko Oyama, Jiro Fukae, Yasushi Shimo, Taku Hatano, Sumihiro Kawajiri, Yasuyuki Okuma, Yutaka Machida, Hideto Miwa, Chikako Suzuki, Asuka Kazama, Masahiko Tomiyama, Takeshi Kihara, Motoyuki Hirasawa, Hideki Shimura, Nobutaka Hattori

**Affiliations:** 1 Department of Neurology, Juntendo University Koshigaya Hospital, Fukuroyama 560, Koshigayashi, Saitama 343-0032 Japan; 2 Department of Neurology, Juntendo University School of Medicine, Tokyo, Japan; 3 Department of Neurology, Abe Neurological Clinic, Iwate, Japan; 4 Department of Neurology and Gerontology, Iwate Medical University, Iwate, Japan; 5 Department of Neurology, Research Institute for Brain and Blood Vessels-Akita Hospital, Akita, Japan; 6 Department of Pathophysiological Laboratory Sciences, Nagoya University Graduate School of Medicine, Aichi, Japan; 7 Brain and Mind Research Center, Nagoya University Graduate School of Medicine, Aichi, Japan; 8 Department of Neurology, Kitano Hospital, The Tazuke Kofukai Medical Research Institute, Osaka, Japan; 9 Department of Neurology, Fukuoka University, Fukuoka, Japan; 10 Department of Neurology, Juntendo University Shizuoka Hospital, Shizuoka, Japan; 11 Department of Neurology, Juntendo University Nerima Hospital, Tokyo, Japan; 12 Department of Diagnostic Radiology, Department of Molecular Medicine and Surgery, Karolinska University Hospital, Karolinska Institute, Stockholm, Sweden; 13 Nozomi Hospital, Saitama, Japan; 14 Department of Neurology, Aomori Prefectural Central Hospital, Aomori, Japan; 15 Department of Neurology, Rakuwakai Otowa Rehabilitation Hospital, Kyoto, Japan; 16 Department of Neurology, Tokyo Rinkai Hospital, Tokyo, Japan; 17 Department of Neurology, Juntendo University Urayasu Hospital, Chiba, Japan

## Erratum

After publication of the original article [1], it came to the authors’ attention that there was an error in the number of male and female participants. The correct number is 90 male and 88 female, not 89 male and 89 female as originally reported.

This error was present in the Methods section of the abstract and in Table 1.

**Table 1:** The baseline characteristics of the study participants

	Mean	Std. Deviation
Age	64.2	9.2
Disease duration	6.8	4.5
Sex (male/female) n	90	88
Wearing off (+/−) n	58	120
Dyskinesia (+/−) n	42	136
Levodopa mg	344.1	202.8
Levodopa (+/−) n	154	24
Dopamine agonist levodopa equivalent dose (mg) [12]	166.3	121.1
Total levodopa equivalent dose (mg) [12]^a^	592.0	317.6
Unified Parkinson’s Disease Rating Scale		
Part I	0.7	1.2
Part II	5.6	3.9
Part III	15.7	8.4
Part IV	1.7	2.2
Total	23.7	11.8
Modified Hoehn and Yahr stage	2.1	0.6
Parkinson’s disease Questionnaire-39		
Mobility	10.4	9.1
Activities of daily living	5.2	5.1
Emotional well-being	6.0	4.4
Stigma	3.2	2.8
Social support	1.4	1.9
Cognitions	4.0	3.0
Communication	1.7	2.2
Body discomfort	2.5	2.6
Total	34.4	24.2

^a^not including istradefylline and zonisamide
